# Biomarkers Analysis and Clinical Manifestations in Comorbid Creutzfeldt–Jakob Disease: A Retrospective Study in 215 Autopsy Cases

**DOI:** 10.3390/biomedicines10030680

**Published:** 2022-03-16

**Authors:** Nikol Jankovska, Robert Rusina, Jiri Keller, Jaromir Kukal, Magdalena Bruzova, Eva Parobkova, Tomas Olejar, Radoslav Matej

**Affiliations:** 1 Department of Pathology and Molecular Medicine, Third Faculty of Medicine, Charles University and Thomayer University Hospital, 140 59 Prague, Czech Republic; nikol.jankovska@ftn.cz (N.J.); magdalena.bruzova@ftn.cz (M.B.); eva.parobkova@ftn.cz (E.P.); tomas.olejar@ftn.cz (T.O.); 2 Department of Neurology, Third Faculty of Medicine, Charles University and Thomayer University Hospital, 140 59 Prague, Czech Republic; robert.rusina@lf3.cuni.cz; 3 Department of Neurology, Third Faculty of Medicine, Charles University and University Hospital Kralovske Vinohrady, 100 34 Prague, Czech Republic; jiri.keller@lf3.cuni.cz; 4 Department of Radiology, Na Homolce Hospital, 150 00 Prague, Czech Republic; 5 Faculty of Nuclear Sciences and Physical Engineering, Czech Technical University, 115 19 Prague, Czech Republic; jaromir.kukal@fjfi.cvut.cz; 6 Department of Pathology, First Faculty of Medicine, Charles University and General University Hospital, 128 00 Prague, Czech Republic; 7 Department of Pathology, Third Faculty of Medicine, Charles University and University Hospital Kralovske Vinohrady, 100 34 Prague, Czech Republic

**Keywords:** Creutzfeldt–Jakob disease, comorbid neuropathology, Alzheimer’s disease, tauopathy, MRI, beta-amyloid

## Abstract

Creutzfeldt–Jakob disease (CJD), the most common human prion disorder, may occur as “pure” neurodegeneration with isolated prion deposits in the brain tissue; however, comorbid cases with different concomitant neurodegenerative diseases have been reported. This retrospective study examined correlations of clinical, neuropathological, molecular-genetic, immunological, and neuroimaging biomarkers in pure and comorbid CJD. A total of 215 patients have been diagnosed with CJD during the last ten years by the Czech National Center for Prion Disorder Surveillance. Data were collected from all patients with respect to diagnostic criteria for probable CJD, including clinical description, EEG, MRI, and CSF findings. A detailed neuropathological analysis uncovered that only 11.16% were “pure” CJD, while 62.79% had comorbid tauopathy, 20.47% had Alzheimer’s disease, 3.26% had frontotemporal lobar degeneration, and 2.33% had synucleinopathy. The comorbid subgroup analysis revealed that tauopathy was linked to putaminal hyperintensity on MRIs, and AD mainly impacted the age of onset, hippocampal atrophy on MRIs, and beta-amyloid levels in the CSF. The retrospective data analysis found a surprisingly high proportion of comorbid neuropathologies; only 11% of cases were verified as “pure” CJD, i.e., lacking hallmarks of other neurodegenerations. Comorbid neuropathologies can impact disease manifestation and can complicate the clinical diagnosis of CJD.

## 1. Introduction

Creutzfeldt-Jakob disease (CJD), the most common human prion disorder [1], is neuropathologically characterized by spongiform encephalopathy involving the subcortical grey matter of the cerebral and cerebellar cortex [2]. It can also be described as encephalopathy with protease-resistant prion protein (PrP) immunoreactivity in the form of plaques, diffuse synaptic, and a patchy/perivacuolar pattern [2].

Three types of CJD are distinguished based on different etiologies [3]: in most countries, sporadic (sCJD) is the dominant form, followed by genetic (gCJD) and acquired CJD, which has two additional subtypes, i.e., iatrogenic (iCJD) [4] and variant (vCJD) [5]. A worldwide incidence of 1–2 cases of sCJD per million inhabitants is commonly reported [6]. The situation in the Czech Republic is similar, but with a slightly higher proportion of genetic cases [7].

Clinical manifestation (criteria for possible CJD) typically includes dementia with pyramidal and/or extrapyramidal signs, cerebellar ataxia, visuospatial dysfunction, myoclonus, and akinetic mutism [8]. Typical biomarkers can help establish the final clinical diagnosis: positive 14-3-3 protein in the cerebrospinal fluid (CSF), generalized periodic EEG patterns, MRI hyperintensities on FLAIR/DWI sequences in the basal ganglia (putamen and caudate) or cortical areas, i.e., cortical ribboning (criteria for probable CJD) and positive results from CSF real-time quaking-induced conversion (RT-QuIC) analysis [8]. Definite CJD is confirmed by neuropathological and immunohistochemistry examination of brain tissue [8].

CJD has long been considered a homogeneous clinical-neuropathological entity. There is, however, increasing evidence of a frequent co-occurrence of other neurodegenerative diseases in CJD cases. Kovacs et al. already published data on the relatively high incidence of tau co-pathology in CJD [9], while Rossi et al. monitored comorbid cases of CJD and Alzheimer’s disease (CJD/AD) and CJD with primary age-related tauopathy (CJD/PART) [10].

The aim of our study was to retrospectively analyse neuropathological findings from autopsy specimens in a large nationwide study of definite CJD cases collected over ten years by the Czech National Center for Human Prion Disorder Surveillance and compare them to clinical, radiological, genetic, and biochemical data. We hypothesised that neuropathological comorbidities could be more frequent than previously thought and could impact disease manifestation or neuroimaging/biochemical results. To our best knowledge, this is the first comprehensive study comparing non-comorbid to comorbid CJD cases based on clinical-neuropathological correlations.

## 2. Materials and Methods

Postmortem confirmed CJD cases resulting from ten years of systematic prion surveillance and available clinical data, neuroimaging findings, and results of neuropathological, molecular-genetic, and immunological investigations were analysed with statistical comparison of non-comorbid versus comorbid cases.

### 2.1. Patients

A total of 215 patients diagnosed with definite CJD (age range 40–87 years, median 66 years) using current diagnostic criteria were neuropathologically examined, and the presence of PrP^Sc^ in brain tissue was confirmed by both western-blot and immunohistochemical methods. The genetic screening revealed 193 sporadic and 22 genetic cases that mostly had the E200K mutation in the *PRNP* gene, but the *D178* and *P102L* mutations and five octapeptide repeat insertions were also found. Clinical as well as neuroimaging data (CT or MRI) from all patients were analysed; moreover, in most cases, EEG and CSF findings were also available.

Characteristics of individual subgroups of pure or comorbid CJD are summarised in Table 1. The table contains detailed information on age, gender, codon 129 methionine and/or valine polymorphism, eventual *PRNP* mutation, specification of the type (type 1 or 2) of abnormal PrP^Sc^ isoform, protein 14-3-3 positivity, CSF neurodegenerative biomarkers (h-tau, p-tau, and Aβ), clinical data regarding dementia, pyramidal and/or extrapyramidal signs, visuospatial or cerebellar dysfunction, myoclonus, and akinetic mutism. Supplementary Material also contains EEG and MRI findings. The data were analysed with respect for patient privacy and with the consent of the local Ethics Committee of the Institute of Clinical and Experimental Medicine in Prague and Thomayer University Hospital, No G-19-18, obtained 26 June 2017.

### 2.2. Tissue Samples

Brain tissue samples were fixed for 3–4 weeks in buffered 10% formalin. Selected tissue blocks, using a standardized protocol [11], were then embedded in paraffin using an automatic tissue processor. Five-μm-thick sections were prepared and stained with hematoxylin-eosin, Klüver-Barrera, and silver impregnation methods. Thirty-six representative blocks from standardized regions were chosen for analysis.

### 2.3. Immunofluorescence and Immunohistochemistry

Briefly, 5-μm-thick sections of formalin-fixed and paraffin-embedded tissue samples were deparaffinized and then incubated with primary antibodies for 20 min at room temperature. For Aβ and PrP^Sc^ antibody staining, 96% formic acid was applied prior to the primary antibody. A second layer for light microscopy visualization, consisting of secondary horseradish peroxidase-conjugated antibody (EnVision FLEX/HRP, Dako M822, Glostrup, Denmark), was applied for 20 min at room temperature. The samples were then incubated with DAB (Substrate-Chromogen Solution, Dako K3468, Glostrup, Denmark) for 10 min to visualize the reaction. Mayer’s Hematoxylin Solution was used as a counterstain.

For confocal microscopy, secondary antibodies conjugated to Alexa Fluor^®^ (see below) were used. Paraffin sections were also treated with 20× TrueBlack^®^ (Biotium 23007, Fremont, CA, USA) diluted in 1× 70% alcohol to quench lipofuscin autofluorescence.

#### 2.3.1. Primary Antibodies

For immunohistochemistry, 5-µm-thick sections of formalin-fixed and paraffin-embedded tissue were selected from the hippocampal region, including the entorhinal and transentorhinal cortex. These were incubated with primary antibodies against the following antigens: (1) PrP (1:8000, mouse monoclonal, clone 12F10; Bertin Pharma A03221, Bordeaux, France), (2) PrP (1:3000, mouse monoclonal, clone 6H8; Prionics 7500996, Schlieren, Switzerland), (3) Aβ (1:1000, mouse monoclonal, clone 6F/3D; Dako M0872, Glostrup, Denmark), (4) Phospho-Tau (Ser202, Thr205) Monoclonal Antibody (1:500, mouse monoclonal, clone AT8; Thermo Fisher Scientific MN1020, Waltham, ME, USA), (5) Ubiquitin (1:500, rabbit polyclonal; Dako Z0458, Lakeside, UK), (6) Phospho TDP-43 (1:4000, mouse monoclonal, clone 11-9; Cosmo Bio TIP-PTD-M01, Carlsbad, CA, USA), (7) Alpha-Synuclein (1:1000, mouse monoclonal, clone 5G4; Dianova NDG-76506, Barcelona, Spain).

#### 2.3.2. Secondary Antibodies

Detection of immunostaining was carried out using horseradish peroxidase–diaminobenzidine (see above) for immunohistochemistry and secondary antibodies conjugated with Alexa Fluor^®^ 488 (1:1000, donkey anti-rabbit, H + L IgG, Thermo Fischer Scientific, Waltham, ME, USA) and Alexa Fluor^®^ 568 (1:1000, donkey anti-mouse, H + L IgG, Thermo Fischer Scientific, Waltham, ME, USA) for immunofluorescence staining. Slides incubated with only the secondary antibody were used as specificity controls.

### 2.4. Microscopy Evaluation

#### 2.4.1. Light Microscopy

Samples were examined, and the results of immunohistochemical methods were classified according to currently valid neuropathological criteria for individual neurodegenerative diseases. Moreover, Alzheimer’s disease was subsequently scored using the National Institute on Aging–Alzheimer’s Association (NIA-AA) consensus scheme [12,13], and dementia with Lewy bodies (DLB) using the DLB consensus criteria [14] with a determination of the Braak stage [15].

#### 2.4.2. Confocal Microscopy

Co-expression of pathogenic protein aggregates was imaged using a Leica TCS SP5 confocal fluorescent laser scanning microscope (Leica Microsystems Inc., Wetzlar, Germany). The HCX PL APO objective was chosen with 60× magnification and an oil immersion pinhole of 1 AU. Anti-rabbit donkey IgG secondary antibody was conjugated to Alexa Fluor^®^ 488 and excited at 488 nm from a 65 mW multi-line argon laser, whereas anti-mouse donkey IgG conjugated to Alexa Fluor^®^ 568 donkey was excited at 561 nm from a 20 mW DPSS laser.

### 2.5. Immunological Methods

#### 2.5.1. CSF Analysis

After a single lumbar puncture and collection, CSF samples were centrifuged at 5000 RPM for 5 min and stored in polypropylene tubes at −80 °C in aliquots to avoid thawing and refreezing until the analysis.

The presence of protein 14-3-3 beta (14-3-3β) was determined using a standardized western blot protocol (adapted from Green et al. [16]) and EURO-CJD standards, with stringent control quality. Briefly, samples in doublets were separated using sodium-dodecyl sulfate-polyacrylamide gel electrophoresis (SDS-PAGE) and blotted onto nitrocellulose membranes. For detection of the 14-3-3β, polyclonal antibody K-19 (1:1000, cat. #sc-629; Santa Cruz Biotechnology, Santa Cruz, CA, USA), and after being discontinued (in 2017), monoclonal antibody B-8 (1:1000, cat. #sc-133233; Santa Cruz Biotechnology) were used. Incubation with an appropriate secondary antibody (1:1000, cat. #sc-2004; Santa Cruz Biotechnology, later with the change of primary antibody cat. #sc-516102; Santa Cruz Biotechnology) was followed by chemiluminescent detection (Pierce ECL Plus Western Blotting Substrate; cat. #32132; Thermo Scientific). A weak positive test was interpreted to mean that one sample load was positive and the other negative (the positive control was always positive). CSF levels of t-tau, p-tau, and Aβ_42_ were measured during routine diagnostic testing using commercially available enzyme-linked immunoassay (ELISA) kits (INNOTEST hTAU Ag, cat. #80323/81572, INNOTEST PHOSPHO-TAU (181P), cat. #80317/81574, INNOTEST β-AMYLOID (1–42), cat. #80324/81576, all Innogenetics/FUJIREBIO); all testing was conducted according to the manufacturer’s protocol. Although the individual values of analytes in the CSF are not entirely decisive and combinations of their ratios would be more accurate [17], the values are as follows. For t-tau, the cut-off was assessed to be 1160 pg/mL with sensitivity of 90.3 % and specificity of 90.7% (AUC: 0.926, *p* < 0.0001). For p-tau and Aβ_42_, the indicative normative values were determined. The values of p-tau levels > 60 pg/mL and Aβ_42_ levels < 430 pg/mL were considered as abnormal. Our laboratory has extensive experience determining CSF biomarkers and successfully participates in the Alzheimer’s Association’s external quality control program. 

#### 2.5.2. Brain Tissue Analysis

Native brain tissue samples were frozen at −80 °C until analysis. The presence of PrPSc was determined using a standardized western blot protocol (adapted from Collinge et al. [18]). Briefly, brain homogenates were treated with Proteinase K (Proteinase K from Tritirachium album, cat. #SRE0005; Sigma-Aldrich, St. Louis, MI, USA). Samples were separated using SDS-PAGE and blotted onto nitrocellulose membranes. For detection of the PrPSc, two different monoclonal antibodies: 12F10 (1:1667, cat. #A03221.200; Bertin Bioreagent, Montigny le Bretonneux, France), and 6H4 (1:5000, cat. #01-010 and since 2017 cat. #7500996, Prionics, Zürich, Switzerland) were used. Incubation with an appropriate secondary antibody (1:2500 and 1:7500, respectively, cat. #P0447; Dako) was followed by chemiluminescent detection (Pierce ECL Plus Western Blotting Substrate, cat. #32132; Thermo Scientific, Waltham, MA, USA). After limited proteolysis, three PrPSc glycoforms were detected.

### 2.6. Molecular-Genetic Methods

*PRNP* (NC-000020.11) is a 16 kb long gene located on chromosome 20 (4686151–4701588). It contains two exons, and exon 2 carries the open reading frame, which encodes the 253 amino acids (AA) PrP protein. Exon 1 is a noncoding exon, which may serve as a transcription initiation site. Post-translational modifications result in removing the first 22 AA N-terminal fragments (NTF) and the last 23 AA C-terminal fragments (CTF).

#### 2.6.1. Study Population

Our study was designed as a retrospective. We analysed data from 215 patients (n = 215), age range 40–87 years, median 66 years. No family history of CJD was retraced. We included patients with postmortem confirmed sCJD and then collected data regarding clinical presentation, biochemical analysis, EEG, and neuroimaging. 

Gene analysis was performed at the level of genomic DNA, assuming an effect on the protein sequence. Only the coding part of the *PRNP* (NM_000311) gene and the adjacent intronic region were evaluated. Genetic analysis of genes was performed from autoptic samples of definitively confirmed cases. DNA was isolated from bone marrow (QIAamp DNA Kits).

#### 2.6.2. Genetic Screen

Mutation analyses by *PRNP* gene sequencing were performed on genomic DNA extracted from bone marrow. The targeted gene captured all exons and the flanking intronic regions of the *PRNP* gene to cover the splice sites. 

Genomic DNA was amplified using two pairs of specific PCR primers (*PRNP* 1F: 5′ TACCATTGCTATGCACTCATT 3′, *PRNP* 1R: 5′GTCACTGCCCGAAATGTATGA 3′ *PRNP*, 2F: 5′AGGTGGCACCCACAGTCAGT 3′ PRNP 2R: 5′ CCTATCCGGGACAAAGAGAGA 3′). Primers were designed using mPCR software, and specific target regions were amplified using PCR (temperature profile in the *PRNP* 1: 95 °C/12 min, 29× (95 °C/30″, 53.1 °C/30″, 72 °C/40″), 72 °C/5′, 4 °C/∞. Temperature profile in the *PRNP* 2: 95 °C/12 min, 30× (95 °C/30″, 60.5 °C/30″, 72 °C/70″), 72 °C/5′, 4 °C/∞). PCR products were enzymatically purified using recombinant Shrimp Alkaline Phosphatase (rSAP) and Exonuclease I (Exo I). The purified products were amplified in a sequencing reaction (temperature profile: 96 °C/60″, 25× (96 °C/10″, 50 °C, 5″, 60 °C/30″), 4 °C/∞) using BigDye Terminator v3.1 Cycle Sequencing Kit’s (Applied Biosystems™). Cleanup PCR products were sequenced on an Applied Biosystems^®^ 3130 Genetic Analyser (using the DNA sequencing Standard Operating Protocol SOPV. We use Sequencing Analysis 5.3.1 software (Applied Biosystems, Waltham, MA, USA—Life Technologies), SeqScape v.2.6 (Applied Biosystems, Waltham, MA, USA—Life Technologies) to evaluate electrophoretic sequencing data.

Results are summarised in Table 2. In our CJD samples, we present an analysis of codon 129 distribution in 215 cases. Methionine homozygotes represented 63.25%, valine homozygotes 15.35%, and methionine/valine 21.40%. 

Although the cohort of 215 patients is limited, we noticed that the ratio of valine homozygotes varied from group to group. In pure CJD, VV homozygotes form 11.00% of cases, in CJD/tau 12.50%, in CJD/AD 20.45%, in AD/FTLD 43.00%, and in CJD with comorbid synucleinopathy 40.00% of cases.

### 2.7. Clinical Data

This study was conceived as a retrospective data analysis. Medical records from different hospitals across the Czech Republic were assessed; in cases with insufficient data, the concerned hospitals were directly contacted to retrieve complete data. For this study, we focused on the presence/absence of key features mentioned by the current WHO diagnostic criteria for probable CJD, i.e., dementia, pyramidal or extrapyramidal signs, visuospatial or cerebellar dysfunction, myoclonus, and akinetic mutism (see Supplementary Material).

### 2.8. Magnetic Resonance Imaging

Available MRI scans were assessed independently by two investigators (J.K. and R.R.) to confirm typical MRI findings listed in the WHO diagnostic criteria for probable CJD, i.e., cortical hyperintensities (typically in the frontal and periinsular areas) and basal ganglia hyperintensities (putamen and caudate) in FLAIR and DWI sequences. DWI data included in all cases an acquisition with a b value equal to 1000. Hyperintensities were evaluated qualitatively, ADC maps were used only to confirm the restriction of the diffusion. No fixed windowing was used as it has been reported to have a lower area under the receiver operating characteristic curves when used by radiologists [19]. Moreover, we used semiquantitative scales for detecting focal atrophy in temporal areas (including the hippocampi) and parietal cortices—for this purpose, we used the MTA scale [20] (measuring mesial temporal atrophy) and the Koedam score [21] (assessing parieto-occipital atrophy). Furthermore, cerebrovascular lesions were coded using the Fazekas scale [22]. All available results are summarised in the Supplementary Material section.

### 2.9. Statistical METHODS

The data set was split into two groups using additional diagnoses; hypothesis testing was performed at *p* = 0.05. Logical explanatory variables were processed using the Fisher exact test of variable independence in 2 × 2 contingency tables to obtain significant Odds Ratios (OR). Real explanatory variables were processed using the two-sampled Wilcoxon–Mann–Whitney test of median equity. All the calculations were performed in the MATLAB 2019 Statistical Toolbox.

## 3. Results

### 3.1. Neuropathological Results

#### 3.1.1. “Pure” CJD

Immunohistochemical methods revealed the presence of several comorbidities in neuropathological examinations. Of the 215 patients, only 24 cases (11.16%) had pure CJD, i.e., lacking any other pathological intra- or extracellular aggregates. The age range of the pure CJD cases was 40–78 years, and the median age was 60 years, which is statistically significantly younger than in the comorbid subgroups.

#### 3.1.2. Comorbid CJD + Tauopathy

Based on clinical data, patients with neuropathological signs of tauopathy lacking clinical correlate were included in this group. The criteria met one hundred thirty-five patients (62.79%), of which 99 (46.05% of 215 cases) had primary age-related tauopathy (CJD/PART). Another 34 cases (15.81%) suffered from CJD/AGD; one patient had CJD/ARTAG (0.47%). Eighteen cases (8.37%) had a combination of CJD/PART and another tauopathy: 13 cases (6.04%) had argyrophilic grain disease (CJD/PART + AGD), and five cases (2.33%) had ageing-related tau astrogliopathy (CJD/PART + ARTAG). Two patients (0.93%) had a combination CJD/ARTAG+AGD, and finally, there was one case (0.47%) with CJD/PART + ARTAG + AGD. The age range of CJD/tau comorbid cases ranged from 49 to 87 years, and the median age was 65 years.

#### 3.1.3. Comorbid CJD/AD

The cohort contained 44 comorbid cases (20.47%) of CJD/AD with possible additional co-pathology. According to the revised “ABC” classification of the National Institute on Aging–Alzheimer’s Association (NIA-AA) [13], changes were classified as level “none” (1 case; 0.47%), “low” (23 cases; 10.70%), or “intermediate” (19 cases; 8.84%), no patients in the “high” category were found. The presence of tauopathies was relatively common with the Alzheimer’s pathology—ARTAG was diagnosed in 10 cases (4.65%), AGD in four cases (1.86%); in one of these cases (0.47%), the criteria for ARTAG + AGD was met. The age range for this group was aged 56–85 years, and the median age was 71 years.

#### 3.1.4. Comorbid CJD/FTLD

Considering comorbid cases of CJD and frontotemporal lobar degeneration (CJD/FTLD) with characteristic frontotemporal clinical symptomatology, seven cases (3.26%) were found, of which six cases (2.80%) had FTLD/tau, and one (0.47%) had frontotemporal lobar degeneration with positive inclusions for ubiquitin-proteasome system markers (FTLD/UPS). The age range was 55–78 years, and the median age was 67 years.

#### 3.1.5. Comorbid CJD + Synucleinopathy

Finally, five patients (2.33%) suffered from CJD with comorbid synucleinopathy; four patients (1.86%) met the criteria for DLB, and one (0.47%) had Parkinson’s disease (PD). The age ranged from 59 to 76 years, with a median age of 71 years.

### 3.2. CSF Analysis Results

CSF analysis was performed antemortem in about 80% of cases: 174 (80.93%) patients were tested for the presence of 14-3-3β, 168 (78.14%) patients were tested for levels of t-tau, p-tau, and Aβ_42_. For CJD, the cut-off level for t-tau was set at 1200 pg/mL [17].

Results of the 14-3-3 analysis are summarised in Table 3 (full details are available in the Supplementary Material). In all groups, 14-3-3β positivity, which is one of the diagnostic criteria for probable CJD (CDC, 2018), was less common than very high t-tau levels (Table 3).

When every group was tested separately, the lowest frequency of 14-3-3β positivity was found in the pure CJD subgroup (12 out of 21, 57.14%). The 14-3-3 positivity was much higher in the comorbidity subgroups (CJD/tau 67 out of 110, 60.91%; CJD/AD 24 out of 33, 72.73%; CJD/FTLD 6 out of 7, 85.71%; and CJD/others 2 out of 3; 66.67%). In pure CJD, t-tau was positive in 17 out of 20 (85.00%). T-tau positivity was higher (CJD/tau 92 out of 105, 87.62% and CJD/AD 31 out of 33, 93.94%) in the comorbidity subgroups.

### 3.3. Clinical Analysis Results

Clinical data were available from all patients and are summarised in Table 1. Diagnostic criteria for possible sCJD [23] were fulfilled in all 215 cases, i.e., all had dementia, and at least two of the four needed signs, i.e., (1) pyramidal or extrapyramidal signs, (2) visuospatial or cerebellar dysfunction, (3) myoclonus, and (4) akinetic mutism. The distribution was as follows: pyramidal or extrapyramidal signs 189 cases (87.90%), visuospatial signs 159 cases (73.95%), myoclonus 133 cases (61.86%), and akinetic mutism 99 cases (46.05%).

### 3.4. MRI Results

**Figure 1:**
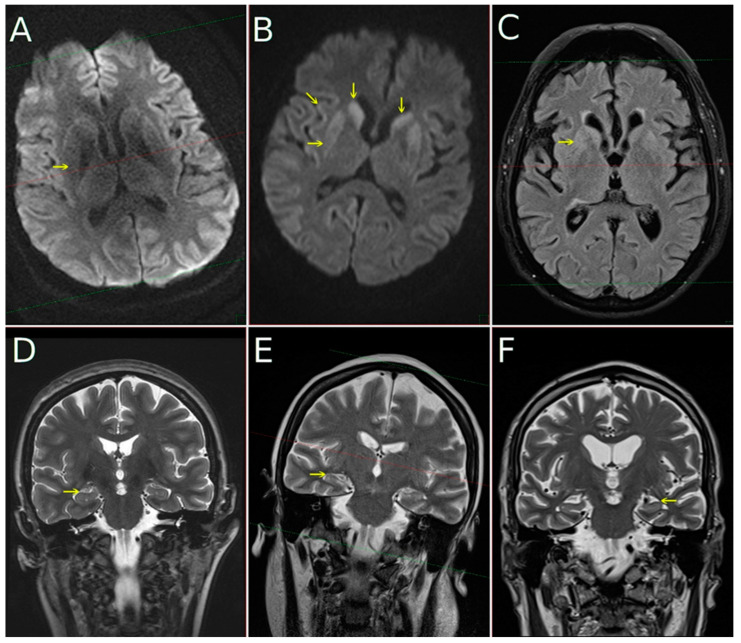
MRI in CJD subjects performed at 1.5T field strength: diffusion-weighed images ((**A**,**B**), DWI) with b-factor 1000, FLAIR image (**C**), coronal T2-weighted images (**D**–**F**). In the first column (**A**,**D**) data from subject with pure CJD—no DWI hyperintensity in putamina (arrow) is present and MTA is 0 (read as normal). In the second column (**B**,**E**) subject with tau comorbidity with mild hippocampal atrophy (MTA 1, arrow on (**E**)) and DWI (**B**) hyperintensity in putamina (horizontal arrow), caudates (vertical arrows) and with cortical ribboning (oblique arrow). In the third column (**C**,**F**) subject with CJD and AD comorbidity is shown. A moderate hyperintensity is visible not only on DWI (not shown), but as well on FLAIR image (**C**). Hippocampal atrophy is well pronounced, MTA 2 (arrow from right side of the (**F**), pointing to the left hippocampus which manifests clear atrophy).

MRIs were available in 206 cases (95.81%), and FLAIR/DWI sequences were available in 188 of these (87.44%; for detailed information, see Table 1). Typical FLAIR and DWI findings meeting the WHO diagnostic criteria for probable CJD were found as follows: cortical hyperintensities in 146 of 188 cases (77.66%), basal ganglia (caudate and putaminal) hyperintensities in 122 of 188 cases (64.89%), and both cortical plus basal ganglia hyperintensities in 109 of 188 cases (57.98%). Manifest mesial temporal atrophy (Scheltens MTA score 2) was present in 32 of 206 cases (15.53%), severe parieto-occipital atrophy (Koedam score 2) was present in 22 of 206 cases (10.68%), and potentially clinically relevant ischemic subcortical white matter lesions (Fazekas score 2 and 3) were present in 32 of 188 cases (17.02%). For detail see Figure 1.

### 3.5. Confocal Microscopy Colocalization Results

**Figure 2:**
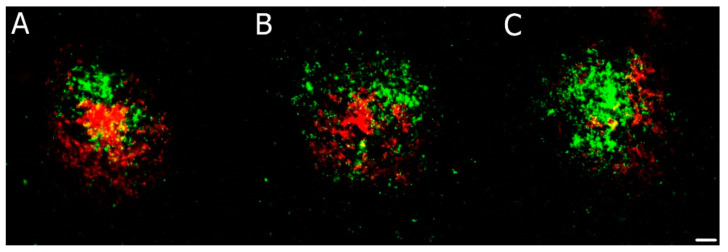
(**A**–**C**) Immunofluorescence illustration of different patterns of amyloid β (Aβ; red) and prion protein (PrP^Sc^; green) colocalization in compound plaques in comorbid Alzheimer’s (AD) and Creutzfeldt–Jakob diseases (CJD) cases. Primary antibodies: anti-PrP (rabbit recombinant monoclonal antibody) + anti-amyloid β-protein (mouse monoclonal antibody). The secondary antibody was conjugated with either Alexa Fluor^®^ 488 (anti-rabbit IgG; green) or Alexa Fluor^®^ 568 (anti-mouse IgG; red). Scale bar indicates 10 μm. Images come from the hippocampal region (archicortical parts).

**Figure 3:**
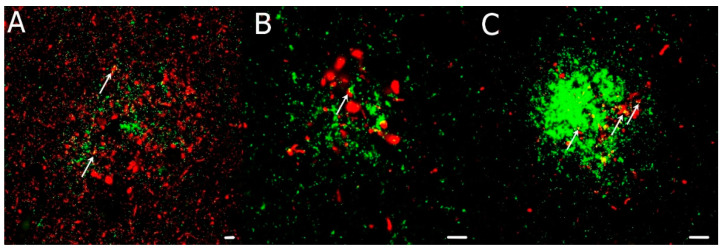
(**A**–**C**) Immunofluorescence illustrates h-tau-positive (red) dystrophic neurites colocalizing with PrP^Sc^ (green) extracellular deposits in comorbid CJD/AD cases. Primary antibodies: PrP (rabbit recombinant monoclonal antibody) + AT8 (mouse monoclonal antibody). The secondary antibody was conjugated with either Alexa Fluor^®^ 488 (anti-rabbit IgG, green) or Alexa Fluor^®^ 568 (anti-mouse IgG, red). Scale bars indicate 10 μm. Arrows indicate minor colocalization of AT8 with PrP. Images come from the hippocampal region (archicortical parts).

**Figure 4:**
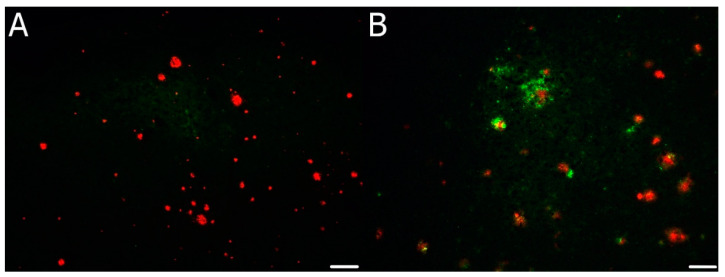
(**A**,**B**) Immunofluorescence illustration of the predominance of non-compound or minimal-compound plaques with minimal colocalization of Aβ (red) and PrP^Sc^ (green) in the majority of plaques. Primary antibodies: anti-PrP (rabbit recombinant monoclonal antibody) + anti-amyloid β-protein (mouse monoclonal antibody). The secondary antibody was conjugated with either Alexa Fluor^®^ 488 (anti-rabbit IgG; green) or Alexa Fluor^®^ 568 (anti-mouse IgG; red). Scale bars indicate 100 μm. Images come from the hippocampal region (archicortical parts).

Multichannel fluorescence confocal microscopy was used in comorbid CJD case examinations to monitor the colocalization of individual pathological aggregates. We devoted our previous publication [24] to the morphology of colocalizing pathological prion protein and amyloid-beta, as well as pathological tau-positive inclusions. Compound plaques with either Aβ or hyperphosphorylated tau protein (h-tau) in colocalization with PrP^Sc^ were sparse. In contrast, PrP^Sc^ aggregates colocalized predominantly with the non-compact (diffuse) regions of Aβ plaques, and colocalization of h-tau with PrP^Sc^ had a dotted pattern. According to the NIA-Alzheimer’s association guidelines, no association between the micromorphology of plaques and type of colocalization with polymorphism at codon 129, type of PrP^Sc^, and the AD ABC score was found. See Figure 2, Figure 3 and Figure 4.

## 4. Discussion

The main findings from comparing pure CJD with different comorbid subgroups were as follows: (1) pure CJD had a significantly lower age of onset; (2) tau comorbidity was associated with a higher probability of putaminal hyperintensities and had a lower MTA score on MRI; (3) AD comorbidity was associated with a higher age of onset, a lower probability for developing putaminal hyperintensities on MRI, and had significantly lower beta-amyloid levels in the CSF; (4) pure CJD compared to CJD/AD had a lower age at onset and a lower MTA score on MRI; (5) comorbid CJD/tau differed from comorbid CJD/AD by having a lower age of onset, lower MTA scores, and higher beta-amyloid CSF levels, and (6) comorbid co-pathology is not dependent on codon 129 homo/heterozygosity nor the genetic background of CJD.

First, the lower age of onset in “pure” CJD cases could be explained by the absence of age-related changes in the brain tissue. In this subgroup, early-onset prion pathology with rapid disease progression probably leads to death before the development of other comorbidities.

Second, comorbid tauopathies were more likely than in other subgroups associated with putaminal DWI hyperintensities on MRI. It is known that changes in these signals correlate roughly equally with vacuolation and the amount of PrP^Sc^ deposition (and less with astrocytic gliosis) [25]. The reason why this hypersignal is more pronounced in CJD subjects with comorbid tauopathy remains unclear since isolated tauopathies are not associated with similar MRI findings, and putaminal deposition of tau protein is not typical; however, it has been reported [26]. We can, therefore, only hypothesise that in subjects with comorbid CJD and tauopathy, the presence of tau facilitates signal increases on DWI either by tau and prion protein co-occurrence or by putaminal microstructure changes, since no micromorphological differences were visible. Lower MTA scores in the CJD/tau subgroup are even more difficult to understand, as in tau positive FTLD, pronounced frontal and temporal lobe atrophy is a frequent finding, as well as in AD, for which higher MTA scores are characteristic [27].

Third, comorbid AD was linked to a higher age of onset. Again, this is a rather surprising finding; as already discussed above, one would expect neurodegenerative comorbidities to have a significant impact relative to the destruction of brain tissue and thus lead to the earlier onset and faster disease progression. Nevertheless, CJD/AD can be viewed from different angles. Pre-existing AD development is more likely to be present at older ages, and from this point of view, it would not be unexpected that comorbid CJD/AD cases are older on average. In one of our previous publications, based on morphological findings using a multichannel fluorescent confocal microscope, we speculated that Aβ_42_ might be acting as PrP^Sc^ seeds within the brain [24] since PrP-Aβ colocalization predominates in the periphery of plaques where Aβ_42_ is more abundant.

Nevertheless, it is important to emphasise that compound plaques represent only a minority of plaques since PrP and Aβ plaques tended to be, in most cases, located separately or formed “minimal compound” plaques. However, even this view of CJD/AD assumes faster progression in CJD/AD comorbid cases than in “pure” CJD, although the data obtained from our 10-year surveillance did not support such a tendency. MRI findings of putaminal sparing can be hypothesised by reduced basal ganglia involvement and fewer parkinsonian features in non-comorbid AD patients compared to pure FTLD patients. The observed low beta-amyloid levels in CSF were concordant with current biomarker findings in pure AD.

A recently published Italian study [10] showed data similar to our observations: the mean age of the CJD/AD cases (71.07 years for our cohort versus 76.1 years for the Italian patients) is strikingly higher than in pure CJD cases; no link was found between the existence of comorbid AD and disease subtype, prion strain, or *PRNP* genotype; in both cohorts, there were no patients in the “high” level of AD; data from both cohorts showed low Aβ levels in the CSF. There was, however, a surprising difference in the percentage of cases in which AD was reported: 61.5% of cases with CJD/AD reported in Italy compared to only 20.47% in our study.

Fourth, some of the previous findings became more obvious when we compared pure CJD to only comorbid CJD/AD. The difference in age of onset and MTA scores were larger between pure CJD and CJD/AD than when comparing pure CJD to all subgroups. The MTA scale is a widely used scoring system for assessing hippocampal atrophy in AD patients and may also monitor disease progression over time [28,29]. We thus suggest that AD could be present before prion disease in older patients.

Fifth, the role of AD on clinical manifestations and biomarkers in our cases was visible when comparing CJD/tau to CJD/AD subgroups. In line with previously discussed points, higher age of onset, higher MTA scores, and lower beta-amyloid CSF levels were key features of the impact of AD on our cohort. 

Sixth, from the genetic point of view, we found no differences between codon 129 status of the *PRNP* gene or between genetic and sporadic CJD; in line with our pilot study, no prominent abnormalities in the deep genetic analysis of “pure” CJD cases and CJD cases in comorbidity with AD and tauopathies, respectively were seen, nor in the analysis of 15 genes related to the most important neurodegenerative diseases [30]. It has been suggested that increased expression of *Syntaxin-6* (*Stx6*) in the basal ganglia could raise the risk of prion disease, and *Stx-6* deposits have been identified as a risk factor for a 4R-tauopathy and progressive supranuclear palsy. The same study also suggests a possible role for *GAL3ST1* [31] in encoding galactose-3-O-sulfotransferase 1, which affects myelin maintenance. This would be consistent with the finding that sphingolipid metabolism is disrupted early in the pathogenesis of prion disorders in mouse models [32].

Finally, compared to a study by Kovacs et al. [9] that focused on tau pathology in CJD and identified 69.3% of cases with prominent tau co-pathology and an additional 16.0% of cases with discrete non-significant tau-immunoreactive neurites, our percentage (11.16%) of “pure” cases was surprisingly low. A possible explanation for the difference in the percentage of “pure” cases by Kovacs et al. is that they did not discuss other pathological deposits. By contrast, our study revealed cases of rare comorbidities such as CJD/FTLD or CJD/synucleinopathies; some correlations between imaging findings and co-pathologies were also found.

The main limitations of our study were the single-center expertise and the very uneven extent of neuroimaging and clinical data from patients examined in different hospitals. These factors made a more robust analysis of the data and clinical correlations difficult.

## 5. Conclusions

We present results from a large cohort of postmortem confirmed CJD patients (with pure CJD and neurodegenerative comorbidities) with clinical, MRI, CSF, neuropathological, and immunohistochemical data. Our retrospective data analysis found a surprisingly high proportion of comorbid neuropathologies, with only 11% of our cases being pure non-comorbid CJD. These patients were found to have the lowest age of disease onset. 

The most interesting findings from our comorbid subgroup analysis were that tauopathy is linked to putaminal hyperintensity one MRIs and that AD is associated with the age of disease onset, the degree of hippocampal atrophy seen on MRIs (low MTA scores), and the low beta-amyloid levels in the CSF. However, further investigation on a broader spectrum of comorbid neuropathologies is needed before evidence of their impact on the clinical presentation can enter routine practice, especially when new biological and potentially targeted therapies become available for treating specific proteinopathies.

## Figures and Tables

**Table 1:** Summary of available epidemiological, neuropathological, immunological and genetic data in non-comorbid vs. comorbid CJD cases. The first column shows the neuropathological diagnosis of patients, second shows the total number of cases in each group, third indicate the gender distribution (female/male). In the fourth column is age range with median age (in years) and the last two columns show methionine/valine polymorphism and presence of 14-3-3 protein in cerebrospinal fluid examinated by western blot.

Diagnosis	No. of Cases	Sex	Age (Years)	Etiology	Genotype	14-3-3 Protein in CSF
CJD	24	24× F	40–78 (median 60)	21× sCJD 3× gCJD	17× MM 4× MV 3× VV	17× positive 7× negative
CJD/tau	135	73× F 62× M	49–87 (median 65)	119× sCJD 16× gCJD	85× MM 34× MV 16× VV	106× positive 29× negative
CJD/AD	44	24× F 20× M	56–85 (median 71)	41× sCJD 3× gCJD	27× MM 8× MV 9× VV	38× positive 6× negative
CJD/FTLD	7	5× F 2× M	55–78 (median 67)	7× sCJD	4× MM 3× VV	6× positive 1× negative
CJD/synuclein	5	3× F 2× M	59–76 (median 71)	5× sCJD	3× MM 2× VV	4× positive 1× negative

F—female, M—male, MM—Methionine/Methionine polymorphism, MV—Methionine/Valine polymorphism, VV—Valine/Valine polymorphism.

**Table 2:** Distribution of MV polymorphisms in pure and comorbid CJD cases.

Polymorphism	Pure CJD	CJD/tau	CJD/AD	CJD/FTLD	CJD/Synuclein
MM	17	85	27	4	3
VV	3	16	9	3	2
MV	4	34	8	0	0
TOTAL	24	135	44	7	5

VV—Valine/Valine, MM—Methionine/Methionine, MV—Methionine/Valine, total stands for total number of cases in each group.

**Table 3:** Numbers (n) and percentage (%) of positive, low positive, negative and unanalysed results of the presence of 14-3-3β, t-tau levels and the combined presence of 14-3-3β and t-tau protein levels in CSF. Percentage is related to the whole cohort.

		14-3-3β (n)	14-3-3β (%)	t-tau (n)	t-tau (%)	14-3-3β + t-tau (n)	14-3-3β + t-tau (%)
pure CJD	pos	12	5.58	17	7.91	11	5.12
	low pos	1	0.47	N/A	N/A	8	3.72
	neg	8	3.72	3	1.40	2	0.93
	no	3	1.40	4	1.86	3	1.40
CJD/tau	pos	67	31.16	92	42.79	74	34.42
	low pos	16	7.44	N/A	N/A	27	12.56
	neg	27	12.56	13	6.05	9	4.19
	no	25	11.63	30	13.95	25	11.63
CJD/AD	pos	24	11.16	31	14.42	27	12.56
	low pos	5	2.33	N/A	N/A	6	2.79
	neg	4	1.86	2	0.93	0	0.00
	no	11	5.12	11	5.12	11	5.12
CJD/FTLD	pos	6	2.79	7	3.26	6	2.79
	low pos	0	0.00	N/A	N/A	1	0.47
	neg	1	0.47	0	0.00	0	0.00
	no	0	0.00	0	0.00	0	0.00
CJD/synuclein	pos	2	0.93	3	1.40	2	0.93
	low pos	0	0.00	N/A	N/A	1	0.47
	neg	1	0.47	0	0.00	0	0.00
	no	2	0.93	2	0.93	2	0.93

pos = positive; low pos = low positive; neg = negative; no = unanalysed; N/A = not applicable. T-tau cut-off: 1200 pg/mL; lower negative. For the combination of 14-3-3β + t-tau, “pos” means both variables are positive, “low pos” means that one of the variables is positive, and the other is negative.

## Data Availability

The authors confirm that all data underlying the findings are fully available without restriction. All data are included within the manuscript.

## Supplementary Materials

The following supporting information can be downloaded at: https://www.mdpi.com/article/10.3390/biomedicines10030680/s1.
