# Supplementary material for: Biomarkers Analysis and Clinical Manifestations in Comorbid Creutzfeldt–Jakob Disease: A Retrospective Study in 215 Autopsy Cases
Source: Biomedicines. 2022 Mar 16;10(3):680. doi: 10.3390/biomedicines10030680 (PMC8944998; doi:10.3390/biomedicines10030680)
Supplement: Supplementary file 1 [file biomedicines-10-00680-s001.zip › Blots.pdf]

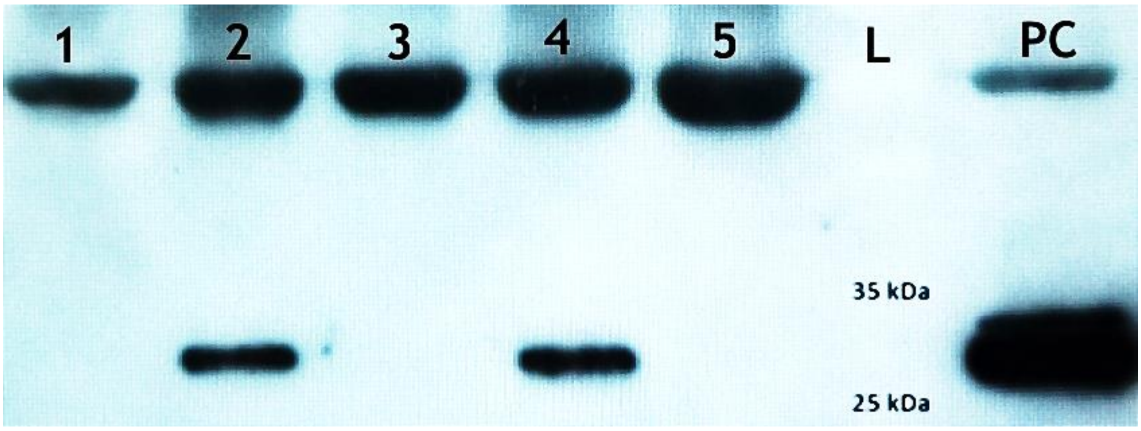

Figure S1: Western blot analysis of 14-3-3 protein. Lane 1, 3, and 5: 14-3-3 protein negative samples, lane 2 and 4: 14-3-3 protein positive samples. Lane 1, 2, and 5: non-CJD positive patients, lane 3 and 4: CJD positive patients. In lane 3 sCJD could be seen results of sCJD patient with type 2 (56-year-old female, MM genotype, neuropathologically sCJD + PART), in lane 4 sCJD with type 1 (71-year-old female, MM genotype, neuropathologically sCJD + AD).

* L – ladder, PC – positive control.

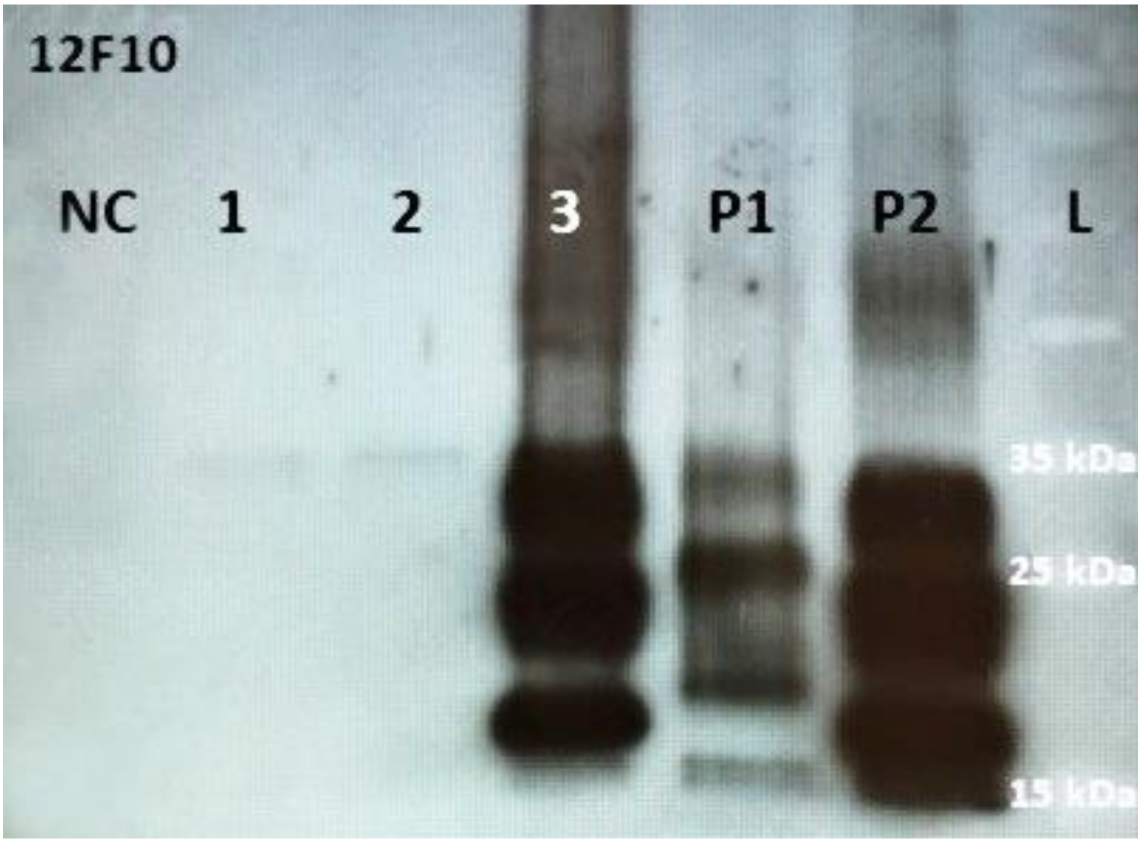

Figure S2: Western blot analysis of PrP. Lane 1 and 2: prion negative samples (cornea donors), lane 3: CJD positive sample (type 2; 43-year old female, MV genotype, neuropathologically sCJD + AGD).

* NC – negative control; P1 – positive control, type 1; P2 – positive control, type 2; L – ladder.
