# Supplementary material for: Biomarkers Analysis and Clinical Manifestations in Comorbid Creutzfeldt–Jakob Disease: A Retrospective Study in 215 Autopsy Cases
Source: Biomedicines. 2022 Mar 16;10(3):680. doi: 10.3390/biomedicines10030680 (PMC8944998; doi:10.3390/biomedicines10030680)
Supplement: Supplementary file 1 [file biomedicines-10-00680-s001.zip › Neuropathological hallmarks.pdf]

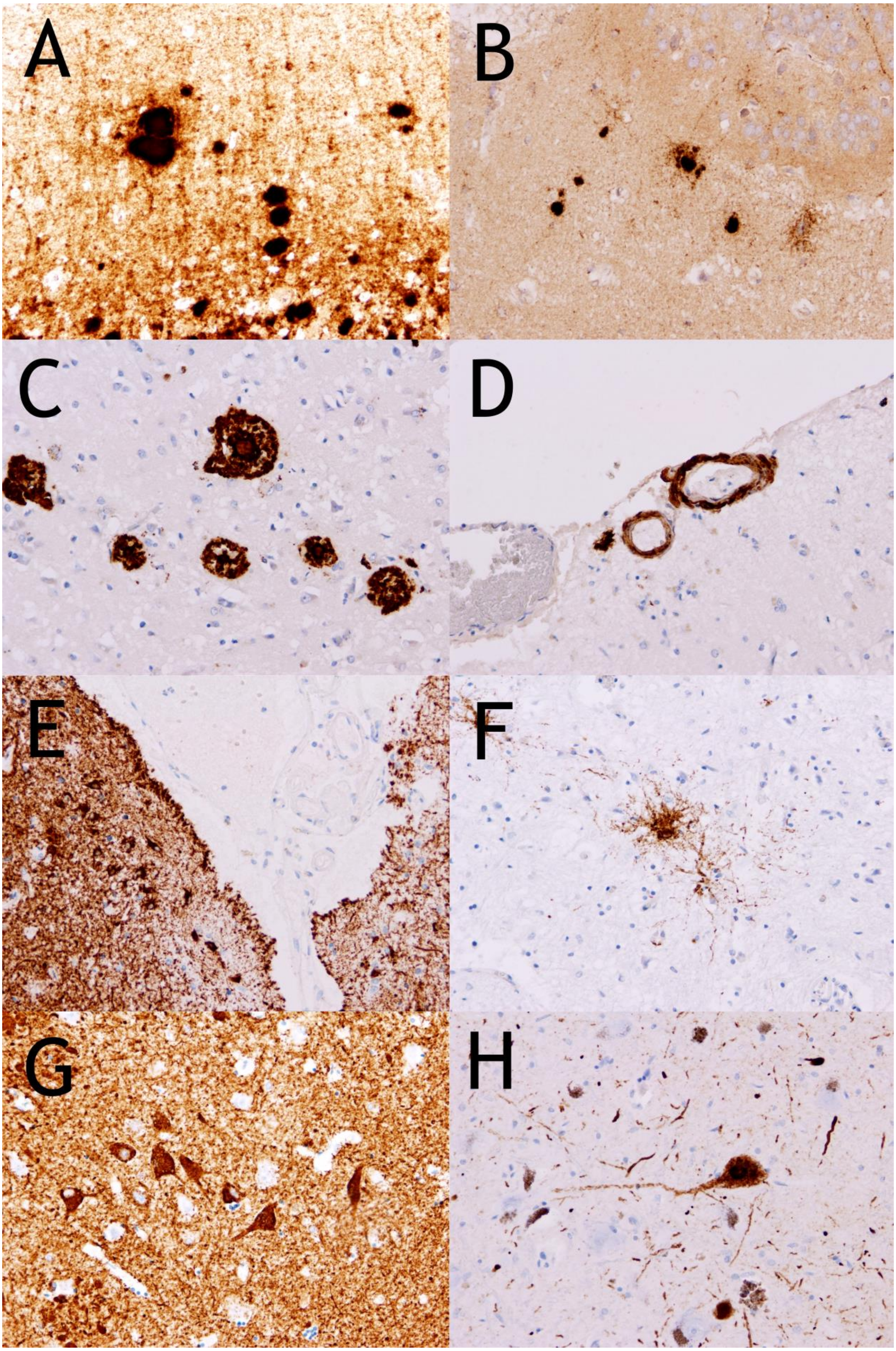

A
B
C
D
E
F
G
H

Figure S3: Typical neuropathological hallmarks of CJD and its comorbid diseases: (A+B) PrP-positive kuru-like plaques may be found in some CJD cases. (C) Extracellular cored neuritic Aβ plaques are characteristic for Alzheimer's disease as well as (D) cerebral amyloid angiopathy. (E+F) Astrocytic inclusions characterize aging-related tau astrogliopathy (magnification 100x and 400x, respectively). (G) Neurofibrillary tangles are crucial for neuropathological diagnosis of primary age-related tauopathy. (H) α-synuclein positive Lewy bodies define Lewy body dementia.
